# Safety and Quality Concerns Regarding Over-the-Counter Sexual Enhancement Products Sold in the USA Market Pose a Major Health Risk

**DOI:** 10.4172/2155-6105.1000299

**Published:** 2016-10-31

**Authors:** Mohammed Ahmed, Suneeta Kumari, Partam Manali, Snezana Sonje, Mansoor Malik

**Affiliations:** Department of Psychiatry and Behavioral Sciences, Howard University Hospital, 2041 Georgia Avenue, NW, Washington, District of Columbia, 20060, USA

**Keywords:** Safety, Sexual, Patient, Behavior

## Abstract

Safety and quality concerns regarding over the counter sexual enhancement products sold in the USA market pose a major health risk to the general public. Nevertheless, the use of herbal medicines continues to expand rapidly across world and many people perceive usage of herbal medication as a safe and reliable way to improve health outcome. The safety of herbal supplements has become a globally major concern in national and international health authorities due to increasing adverse events and adulterations associated with usage of herbal medications. These non FDA approved products with unknown ingredients are widely accessible for purchase ranging from local food, drug stores and to the internet.

These Erectile Dysfunction (ED) pills may contain Sildenafil, the active ingredient of Viagra in much higher quantity then legally prescribed by a licensed physician or they may contain unknown quantities of Thiosildenafil, the active ingredient in Cialis. The types of chemicals found in these medications are making it harder for regulatory authorities to track them down. These products keep the consumer in the dark in terms of the quantity, ingredients, effectiveness and possible side effects. These sexual enhancement products are being sold as safe and natural with false hopes to resolve erectile dysfunction. Patients who are prone to impulsive hypersexual behavior such as patients with bipolar disorder, substance use, borderline personality disorder and those who may feel adamant to discuss erectile dysfunction with their physicians are more likely to become the victims of using illicit medications/ drugs with serious health risks consequences.

We present a case report of an individual with bipolar disorder and hypersexual behavior who became victim to over the counter sexual enhancement products/supplements which caused serious health and life threatening consequences.

## Introduction

Safety and quality concerns regarding over the counter sexual enhancement products sold in the USA market pose a major health risk to the general public. Nevertheless, the use of herbal medicines continues to expand rapidly across world and many people perceive usage of herbal medication as a safe and reliable way to improve health outcome. The safety of herbal supplements has become a globally major concern in national and international health authorities due to increasing adverse events and adulterations associated with usage of herbal medications.

These products are accessible for purchase from local food, drug stores and the internet. These products are marketed as supplements and are not currently under the regulations of the Food and Drug Administration (FDA) and are thus released to the general public containing unknown amounts of medications or drugs that are normally available by prescription only in the USA [1]. It is difficult to analyze the herbal products that cause adverse events due to insufficient information, lack of expertise. Inadequate regulatory measures, weak quality control system and uncontrolled distribution by various vendors are some of the reasons that has augmented herbal supplements market [2].

In recent years the Food Drug Administration FDA has noted an emerging trend where dietary supplements used for sexual enhancements have untested pharmaceutically active ingredients leading to harmful consequences [3].

The FDA has identified more than 350 different supplements marketed for sexual enhancement and found to be tainted with prescription medication of which the quality and quantity is questionable due to lack of oversight in these products. Consumer request has created a huge demand for these products and has led to sales in the hundreds of million to billions of dollars in the US and world-wide [1,3]

This multi-million dollar business is driven by various psychological factors that many men may be too embarrassed to ask their primary care for ED treatment, they may have experienced side-effects after using Sildenafil or Tadalafil or they may have a large co-payment attached to their prescription for erectile medication. Therefore, these patients may turn to using products they deem natural or without side effects and thus believe these products are safer to use. As in the majority of these cases, the manufactures of these products neither list the true content and nature of the ingredients nor indicate the quantity or quality of the substances used in these products [4].

Furthermore according to Fox News report published in November 2007 popping herbal sex pills linked to increased risk of stroke, headaches and vision problems. The pills marketed as safe herbal alternatives to Viagra and other prescription medication for erectile dysfunction exhibits a hidden danger and adverse effects especially among people with pre-existing heart conditions. Concomitant use of sex pills with high blood pressure and heart meds can lead to a stroke or even death [5].

### Case presentation

The patient, 55 year old African American Male (AAM) Piano performer, church minister and former teacher, with PMH of CHF with EF of 50–55% HTN, DM type 2 insulin dependent, CKF stage 3, with past psychiatric history of bipolar disorder presented to Emergency Dept. (ED) of metropolitan hospital in summer with right hemiparesis, unstable gait. The patient reported that he went to bed at 10 pm after taking his insulin (25 units) without checking his blood sugar. Patient woke up at 6 am in his bedroom floor not knowing how he got there with swelling and significant pain in his right arm and hand. He reported no urinary incontinence. Later patient called his friend and request to take him to nearby healthcare facility Patient reported right hemiparesis, unstable gait and imbalance. He was brought to ED in an ambulance by his friend.

### PE

At the time of admission, he presented with slurred speech, symmetric face. Vitals: 90/55 mm Hg, RR 18, HR 77 Temperature 98.8 and O_2_ stat of 98. The finger stick fasting blood sugar was 214 mg/dl. Pt. had right upper extremity (RUE) swelling and bruising. PERRLA, lungs were clear to auscultation bilaterally, 5/5 motor bilateral except right arm 3/5, at hands he had no median nerve function at all, he had good sensation of the small finger and he was able to flex with his ulnar nerve his small finger and to some extent the ring finger. He had no sensation in right arm up to the elbow. He had lost sensation down the center of the forearm of the volar aspect. His Triceps and Biceps mobility was within normal range. Patient had tongue laceration with slight bleeding as a result of tongue biting.

Patient was diagnosed with expressive aphasia, with median nerve palsy, r/o new onset seizure activity, r/o hypoglycemia. Non-enhanced computed tomography (CT scan) of the brain revealed no hemorrhage as well as MRI did not show any acute changes.

During initial assessment, patient reported he has been abusing over the counter erectile dysfunction (ED) pills since last ten (10) years which he purchased over the internet and from the local grocery stores, with the majority being labeled made in China. Those products packaging did not list quantity, ingredients, warning sign or harmful side effects of the products. Per patient “I was not aware of the contents in neither the ED pills nor the substances contained within it”. Unfortunately, patient took several different brands of ED pills at the same time, assuming its “safe” as proclaimed on the packaging. To list some of these products which the patient took, as confirmed by patient, included “Male Enhancement”, “Extenze” which had the word written “doctor approved”. This product as per FDA website has the undeclared ingredient “Sildenafil” and FDA has issued warning Patient reported he is also using “Mojo Risen” which has been shown to contain Noracetildenafil as confirmed by FDA laboratory analysis. This product is structurally similar to Sildenafil the active ingredient in Viagra. This undeclared substance may interact with Nitrate, as does Sildenafil, and can cause a dangerous drop in blood pressure [2].

In the ER he was treated 1 gram of Dilantin and Ativan and he was discharged in a stable condition to his home, he was alert, orientated with no acute distress. The vitals at the time of discharge were B.P 125/60 RR20, HR of 68 Temperature 96.4. Fasting blood sugar level 140 mg/dl. Patient was encouraged do not use over the counter sexual enhancement products; he was referred to primary care provider regarding consultation for erectile dysfunction.

Two weeks after discharge the patient was brought back to the Emergency Department at metropolitan hospital by friend.

He was found unresponsive in his apartment by his home health aide; he had saliva drooling from the mouth, and was unresponsive to questioning from the admitting physician. Patient on admission had GCS of 8/15. In the ER patient had seizure like activity for which he was given 1 g of Dilantin and Ativan. During his hospital stay, it was determined that the patient had suffered a CVA. After stabilization of his symptoms Patient was discharged to a nursing home as he was found incapable of performing ADLs by himself. After one month of discharge patient’s condition did not returned to baseline.

Unfortunately, as reported by patient he went home after discharged and he continued to abuse ED supplements. It was concluded by the psychiatric treatment team that the patient’s physical state/seizures like activity was resulting from his continued misuse of over-the counter- ED supplements as self-reported by patient and his friend.

## Discussion

The availability of these products through the internet and over the counter in local grocery stores places has a huge clinical challenge to health care. Regulatory agencies emphasizing the impact of the usage of these supplements on the health of general public and striving to impose strict regulations on these products.

Nevertheless, FDA faces serious obstacles in identifying these products true ingredients in a timely fashion to prevent physical damage being done to general public. Since these supplements are not regulated by the FDA consumers and vendors considered it safe until proven otherwise.

Concrete steps must be taken to improve the safety and well-being of consumers and to decrease the health related consequences from those products which should only be taken under the advice of a physician.

The Public should be educated at local, state and national levels to avoid miss use of sexual enhancement products purchased through the internet or over-the-counter.

Strict guideline and regulations for erectile dysfunction supplements should be implemented to prevent misuse of medications such as Sildenafil or Tadalafil or medications similar in structure.

Establishment of National and International Databases to better inform the public of the use and consequences of misuse of ED medications and supplements through clear and concise listing of ingredients on product labels.

Implement tougher guidelines and monitoring of products sold by manufacturers and to implement regulatory monitoring on the companies involved in manufacturing of over the counter products.

Physician should have low threshold in prescribing for erectile dysfunction medication and to carefully monitor patients who are at risk of developing ED in the future to encourage more open conversation about this topic and eliminate the stigma of ED.

## References

[1] Food and Drug Administration (2012). Public Notification: “Mojo Nights” contains hidden drug ingredients.

[2] Shaw D, Graeme L, Pierre D, Elizabeth W (2012). Pharmacovigilance of herbal medicines. J Ethnopharmacol.

[3] US Food and Drug Administration (2012). Tainted Supplements CDER.

[4] Canham M (2013). Feds plow new ground in spiked supplement case.

[R5] 5(2017) Popping Herbal Sex Pills Linked to Increased Risk of Stroke, Headaches, Vision Problems.

